# Can cognitive processes help explain the success of instructional techniques recommended by behavior analysts?

**DOI:** 10.1038/s41539-017-0018-1

**Published:** 2018-01-17

**Authors:** Rebecca A. Markovits, Yana Weinstein

**Affiliations:** Department of Psychology, University of Massachusetts Lowell, Lowell, MA 01854 USA

## Abstract

The fields of cognitive psychology and behavior analysis have undertaken separate investigations into effective learning strategies. These studies have led to several recommendations from both fields regarding teaching techniques that have been shown to enhance student performance. While cognitive psychology and behavior analysis have studied student performance independently from their different perspectives, the recommendations they make are remarkably similar. The lack of discussion between the two fields, despite these similarities, is surprising. The current paper seeks to remedy this oversight in two ways: first, by reviewing two techniques recommended by behavior analysts—guided notes and response cards—and comparing them to their counterparts in cognitive psychology that are potentially responsible for their effectiveness; and second, by outlining some other areas of overlap that could benefit from collaboration. By starting the discussion with the comparison of two specific recommendations for teaching techniques, we hope to galvanize a more extensive collaboration that will not only further the progression of both fields, but also extend the practical applications of the ensuing research.

## Introduction

While both behavior analysis (behaviorism) and cognitive psychology study human behavior, they have long been seen as opposing one another. Behavior analysis looks to explain behavior through behavior–environment interactions, whereas cognitive psychology seeks to explain behavior through mental processes. Despite these differences, both areas study methods for improving student performance in the classroom. Furthermore, when it comes to improving educational outcomes, the two fields produce remarkably similar recommendations. Yet, the two fields have not come together to discuss these recommendations.

For the last several decades, behavior analysts have studied human behavior as it relates to learning and subsequent performance. While the majority have focused on treatment of individuals with autism and other developmental disabilities, some have evaluated applications of behavior analytic principles and technologies to K-12 and higher education.^1–7^ Several studies have looked at ways to increase active student responding (ASR), or the observable response a student makes to an instructional antecedent. In other words, ASR increases class participation in a way that helps the students learn more effectively. While there are various different ASR methods, general findings reflect increases in student learning when some ASR methods compared to none.^1,2,6^

Concurrently but independently, cognitive psychologists are investigating the mental processes that may impact learning in an educational context.^8^ Recently, classroom recommendations based on these findings have begun to emerge.^9^ In particular, recent work highlights the benefits of retrieval practice—or quizzing—to long-term learning.^10^ Recommendations made by cognitive psychologists based on this line of inquiry appear to be similar to those made by behavior analysts; and yet, the two fields have never come together to discuss these recommendations. In addition to behavioral explanations, the effective pedagogical practices recommended by behavior analysts may also be explained by theories that are concurrently being tested in the field of cognitive psychology—and vice versa. Here, we explore two teaching techniques recommended by behavior analysts—guided notes and response cards—and the cognitive and behavioral processes that might contribute to the effectiveness of these strategies.

## Guided notes/Generation effect

Guided notes are instructor-prepared notes that cue students to respond to key information in a lecture or discussion. The cues can be blank spaces where students add information, or notations prompting students to engage in a variety of note-taking behaviors (e.g., asterisks next to terms students should define or provide an example for). According to behavior analysis, there are several advantages to using guided notes. First, students leave the lecture with more accurate notes, which particularly helps students who lack good note-taking skills better prepare for exams.^11^ Second, guided notes require that students actively engage with material by completing the notes, which can increase achievement and classroom participation when compared to conditions that present an outline of the lecture and relevant terms, or consist of a lecture only, or lecture plus slides.^12,13^ Guided notes are also thought to help with organization: The notes may help students identify important information, and allow them to review topics prior to class.^11^

Several studies have evaluated the effectiveness of guided notes compared to other forms of note taking, or being provided full copies of instructor notes. A meta-analytic review of the research on guided notes included eight studies, five of which tested school-aged children and three of which tested college-aged students (studies were a mix of male and female students, and some included participants who were diagnosed with a learning disability).^5^ All eight studies, including those with learning-disabled participants, found consistently higher performance on later quizzes when guided notes were used compared to conditions without guided notes. While there is still much work to be done to determine the best method of incorporating guided notes into a course, as well as why guided notes increase performance, the literature so far suggests that guided notes are an effective method for increasing students’ performance in class as well as on quizzes and tests.

The idea that actively producing information (in this case, taking notes rather than receiving full copies of instructor notes) helps learning has also been investigated by cognitive psychologists; they have explored the effects on memory of generating (or producing) information, as compared with just passively reading the same information. In the paper that coined this term, participants either read words, or they generated them from clues (e.g., reading synonym pairs like “rapid-fast”, vs. generating the second word, e.g., “rapid-f”); performance on later memory tests indicated an advantage of generated compared to read information.^14^ This advantage was subsequently named the “generation effect” and has been extensively replicated^15^ and investigated in applied educational settings, such as by having students answer questions^16^ and generate self-explanations^17^ to aid learning.

Several competing cognitive theories have been proposed to explain the generation effect, including increased mental effort^18^ and transfer-appropriate processing.^19^ When comparing guided note-taking to providing students with completed notes, these theories also apply. That is, filling in the guided notes vs. passively listening takes greater mental effort, and filling in the notes is more similar (transfer-appropriate processing) to taking a test than is passive listening. And yet, despite the seminal paper on the generation effect^14^ having over a thousand citations, only one of them examines guided notes,^20^ and even in that paper, only a single sentence refers to the generation effect. A handful of additional papers on guided notes also mention the generation effect,^21^ but do not refer to the foundational paper,^14^ suggesting a disconnect between the two fields.

## Response cards/Retrieval practice

Response cards are another tool that instructors may use to increase ASR during class time. Response cards are typically the size of 3 × 5” index cards with answers on one or both sides. Color coding may be used to help teachers and students discriminate between answers. In one study, students wrote “A” / “True” on a green side of a card and “B” / “False” on the red side.^4^ The instructors then asked questions throughout the lecture that were either true/false statements or involved the students choosing between options A and B. In another study, cards were pre-printed for students with answers that corresponded to questions presented during that day’s lecture.^6^ A third option is to use a small, laminated board on which students write answers to hold up later.^22^ Sometimes, the response cards are used to elicit answers about material just presented, whereas other times, questions refer to information presented in previous classes. Thus, many different forms of this method exist, ranging from immediate multiple-choice questions, to those requiring a delayed, open-ended response.

Studies have shown that student performance on quizzes and tests increases when response cards are used. In one study, response cards were compared to hand raising in a 5th grade classroom; teacher presentation rate, number of student responses, accuracy of student responses, next-day quiz scores, and bi-weekly review test scores were measured.^22^ Even though the rate at which the teacher presented opportunities to respond did not vary greatly between the two conditions, students responded to only 4% of the questions in the hand raising condition, whereas they responded to 68% of the questions in the response card condition. Furthermore, while there was no difference in accuracy of responding across the two conditions, results on the next-day and bi-weekly quizzes showed a marked increase in scores following response cards. Similar results were found with response cards in an undergraduate Psychology of Learning course,^23^ comparing student performance in a traditional lecture with no response cards and a traditional lecture with response cards. Furthermore, there was high social validity for this method: students found response cards helpful in attending to the lecture and reported that they would like more professors to use response cards during lectures. These studies show that not only are response cards a low-cost/low-effort intervention, but they are both effective in increasing student academic performance and acceptable to students.

From the teacher’s perspective, response cards allow them to see what types of errors the students are making and pinpoint what part of the lecture or instruction the students are not understanding. Behavior analysis explains the learning process thus: When a stimulus or context requires a particular response, correct responding is reinforced while incorrect responding is not reinforced. With repeated presentations of the stimulus/reinforcer, correct responding increases and becomes more fluent while incorrect responding decreases. For example, a student might be given a term and asked to define it. Their first attempt may have many errors and lack fluency; students would then receive feedback on how well they did that would allow them to do better the next time. As they practice (come into contact with the combination of the term and the feedback—the antecedent and the consequence) their correctness and fluency increase. Once fluency and mastery has been gained, we say that the term has control over the response of giving the correct definition—or that stimulus control has developed. Now, when they see the term on a test, they are now able to provide a correct definition because that term triggers the correct response. According to behavior analysis, this process is promoted by the use of response cards.

While behavior analysts have been investigating and recommending response cards as a pedagogical tool to aid learning, cognitive psychologists have been studying a similar concept under the term “retrieval practice”. In a typical retrieval practice experiment, participants in one condition study some information, then practice retrieval, followed by a final test. In a control condition, students instead restudy the material before taking the final test. Experiments differ by type of retrieval practice (e.g., writing everything students can remember from memory,^24^ answering a multiple-choice quiz^25^), type of materials studied (e.g., foreign language vocabulary,^26^ prose passages^27^), whether feedback is provided, and more. However, the consistent finding is that performance on a delayed test is better after retrieval practice than after restudy, (cf. a meta-analysis of 217 retrieval practice studies^28^). Retrieval practice (also known as “the testing effect”) describes the idea that bringing information to mind produces learning; proposed mechanisms include reduction of interference,^29^ increased motivation,^30^ and context reinstatement.^31^ Although this effect is highly relevant to the use of response cards, a Google Scholar search reveals no publications about response cards mentioning retrieval practice (or vice versa), although one meta-analysis of audience response systems^32^ does briefly mention both.

## Conclusion

The main difference between cognitive and behavioral research is that cognitive psychologists seek to explain the specific processes in the mind that give rise to observed behaviors (here, better performance on memory tests after generation or retrieval practice than after passive re-reading) while behavioralists focus on manipulating the environment to produce those observed behaviors. Regardless of these differences, both fields want to improve educational outcomes for students through effective pedagogical techniques. To the extent that the two fields appear to be investigating the same types of educational interventions, a more open dialog would be more efficient for the advancement of both fields. This review focused on two behavioral techniques (guided notes and response cards) and two analogous cognitive mechanisms (generation and retrieval practice). Two other possible areas of overlap would be interesting to explore but are beyond the scope of this manuscript. First, mind-wandering^33^ could help explain covert behavioral chains (i.e., internal processes that include both unpredictable trains of thought and systematic problem solving) and competing contingencies (i.e., why we choose to engage in one behavior vs. another given the opportunity to do either). Second, the dual coding theory^34^ could help explain the effectiveness of complex stimulus control, stimulus shaping, and other prompting techniques used in behavior analysis. In addition to exchanging ideas about overlapping techniques and recommendations, both fields may benefit from comparing theories of why those techniques are effective to help inform future research and applications. In fact, such partnerships between the two fields have been achieved in other domains, such as visual attention.^35^ Understanding the mechanisms of effective pedagogical techniques is crucial from a practical standpoint because it enables us to develop better recommendations for teachers who want to implement the techniques in their classrooms.
